# Innovative Apparatus for Testing Filtration, Sorption and CO_2_/CH_4_ Exchange Sorption Processes Under Isobaric Conditions on Sorbent Subjected to Confining Pressure in Terms of Laboratory Tests of CO_2_-ECBM Technology

**DOI:** 10.3390/s20205823

**Published:** 2020-10-15

**Authors:** Mateusz Kudasik, Norbert Skoczylas, Anna Pajdak

**Affiliations:** The Strata Mechanics Research Institute of the Polish Academy of Sciences, Reymonta 27, 30-059 Cracow, Poland; skoczylas@img-pan.krakow.pl (N.S.); pajdak@img-pan.krakow.pl (A.P.)

**Keywords:** exchange sorption, carbon sequestration, ECBM, confining pressure, greenhouse gas, coal

## Abstract

In recent years, the interest in the sorption properties of coal in conditions corresponding to in situ has increased due to the continuous development of research on CO_2_-ECBM (Enhanced Coal Bed Methane recovery) technology. In order to gain a better insight into a number of phenomena related to filtration, sorption and CO_2_/CH_4_ exchange sorption occurring in coal loaded with confining pressure, which corresponds to the in situ conditions, an innovative research apparatus was built to enable temporal and spatial analysis of these phenomena. The constructed apparatus consists of three systems: a high-pressure system, a gas injection system and a gas emission system. The work presents the results of basic apparatus tests, which were aimed at checking its correct operation and determining its specifications. These tests involved carrying out trial measurements of methane (CH_4_) filtration processes, CH_4_ sorption and CO_2_/CH_4_ exchange sorption on a coal sample. The results of the tests showed among other things that the apparatus ensured the regulation of the confining pressure in the range of 0.1–40 MPa, the regulation of the pressure at the inlet and outlet of the sample in the range of 0.1–1.6 MPa and 0.1–1.0 MPa and the measurement of changes in the sample volume in the range of 0–7.85 cm^3^. The results of the tests confirmed the correct functioning of the constructed apparatus.

## 1. Introduction

Metamorphism processes and natural gas migration phenomena in the pore space of a rock mass, as well as gasogeodynamic phenomena and other natural processes occurring deep underground, are extremely complicated and their recognition and reconstruction is a huge challenge for engineers and scientists. Technological progress increases the potential possibilities for conducting scientific research with much greater precision and control, enabling the replication and simulation of in situ conditions for a better understanding of phenomena occurring in the natural environment. In particular, the reconstruction under laboratory conditions of phenomena occurring deep underground in strata has been and still is a huge challenge in the matter of development and better recognition of the Earth’s crust structure.

Progressive climate changes on Earth result in destabilization of a very complex and interlinked system of environmental components that adversely affect the human life. The concentration of carbon dioxide (CO_2_) in the Earth’s atmosphere has remained around 280 ppm for the last 800,000 years. It has been increasing annually by around 1.2–2.0 ppm since the mid-19th century. The atmospheric level of CO_2_ has raised from 280 ppm to 415 ppm in the last 170 years [1]. Due to the emission of CO_2_ and also methane (CH_4_) and nitrogen oxides (NO_x_), the average temperature on Earth has increased by about one Kelvin since the beginning of industrialization. This problem was raised in the latest IPCC (Intergovernmental Panel on Climate Change) report and in the COP24 climate summit in Katowice in 2018. An earlier IPCC (Intergovernmental Panel on Climate Change) SRES (Intergovernmental Panel on Climate Change) report from 2007 touched on the aspect of anthropogenic CO_2_ storage and specified geological structures that are suitable for the application of this technology. One of these structures was a deep, unexploited coal seam, which may be used in CO_2_-ECBM (Enhanced Coal Bed Methane recovery) technology.

One of the parameters that has a decisive influence on the assessment of the possibility of using CO_2_-ECBM technology is the sorption capacity of the coal bed in relation to CH_4_ and CO_2_. The sorption capacity of coal is a parameter that quantifies the ability of coal to sorb gases under certain pressure and temperature conditions. In the sorption equilibrium state of the coal-gas system, approximately 95–98% of the gas is sorption-bound in the coal pore space and the remaining part is free gas [2,3,4]. The value of the sorption capacity of coal, apart from the thermodynamic conditions, is influenced by the coal rank, the maceral composition, the type of gas sorbed and the presence of other gas sorbates and water [5,6]. One of the important parameters that may affect the sorption capacity of coal is also the overburden pressure to which it is subjected. Sorption of gas leads to changes in the structure of coal caused among others by sorption stress and swelling [7,8,9,10,11,12,13,14]. The loading of coal limits its swelling [3,8,15,16]. The confining pressure caused by the loading limits the sorption capacity of coal in relation to both CH_4_ and CO_2_ by up to 80–90% [3,17,18,19,20,21]. The main reasons for the reduction of the sorption capacity as a result of applying pressure on coal seams by the load include pressure-induced changes in structural properties, a reduction of porosity, a limitation of specific surface area availability, a reduction of permeability and diffusivity and a limitation of sorption-induced swelling that causes internal strains and rearrangement of the coal structure [22,23].

The exchange sorption process used in CO_2_-ECBM technology is extremely complicated due to the interaction between CO_2_ and CH_4_ in competitive sorption [24] and diffusion in opposite directions. In addition, coal swelling in the presence of CH_4_ and CO_2_ is different in its magnitude [9,25,26]. As a result, local changes in coal permeability occur during the CO_2_/CH_4_ exchange sorption process.

The CO_2_/CH_4_ exchange sorption tests on coal samples free of stress have been conducted since the eighties of the last century. The paper by Fulton et al. [27] presented the first laboratory tests of CO_2_ injection into coal in order to replace CH_4_. Further research on competitive adsorption/desorption processes was presented in the paper by Reznik et al. [28] where at higher CO_2_ injection pressures (between 3.4 MPa and 5.5 MPa), the entire CH_4_ content was displaced from the coal. Most laboratory tests on CO_2_/CH_4_ exchange sorption include an analysis of the sorption properties of coal as a potential CO_2_ storage reservoir and CH_4_ resource [29,30,31,32,33,34,35]. A detailed analysis of the CO_2_/CH_4_ exchange sorption process was the subject of several works [20,25,36,37,38,39,40,41,42,43,44].

Interest in the sorption properties of coal in conditions corresponding to in situ has been increasing due to the continuous development of research on CO_2_-ECBM technology. This technology was first used in the San Juan Basin, New Mexico, USA in the mid-1990s [45]. However, the coal mined in that area differs significantly in its petrophysical properties from coals from the European basin especially in terms of higher permeability and lower coal rank.

In order to improve the knowledge of processes accompanying the application of CO_2_-ECBM technology, new test stands are being created in research centers to carry out sorption measurements on coal under stress. One of the few laboratory stands enabling the exchange sorption tests on coal under confining pressure conditions has been presented in the article by Wolf et al. [38]. A cylindrical coal sample with a diameter of 72–78 mm and a length of about 200–300 mm, tightly covered with a flexible synthetic cover holder, was placed in a high-pressure chamber where it was compressed using nitrogen (N_2_) or oil as an intermediate pressure medium. The maximum confining pressure was 11 MPa when using N_2_ and 50 MPa when using oil. The maximum sorbate pressure was 1 MPa less than the applied annual pressure. The degassed sample was saturated with CH_4_. An injection of CO_2_ took place on a dry or water saturated coal sample (a water saturation step before injecting CO_2_). At the sample outlet, gas concentration sensors and a flow meter were connected to the high-pressure chamber. Sorption was measured by a volumetric method in non-isobaric conditions.

At the Strata Mechanics Research Institute of the Polish Academy of Sciences, innovative methods and measuring tools for unique research related to the presence and sorption of gases in the rock pore space have been developed over several years [46,47,48,49,50,51,52,53,54]. Studies conducted by the Institute also cover issues related to CO_2_/CH_4_ exchange sorption as well as gas sorption in coal under confining stress conditions [6,43,44,55,56,57]. In order to gain a better insight into a number of phenomena such as filtration, sorption and CO_2_/CH_4_ exchange sorption occurring in coal loaded with confining pressure that corresponds to the in situ conditions, innovative research equipment was built to enable the temporal and spatial analysis of these phenomena.

## 2. Construction of Apparatus

**Figure 1:**
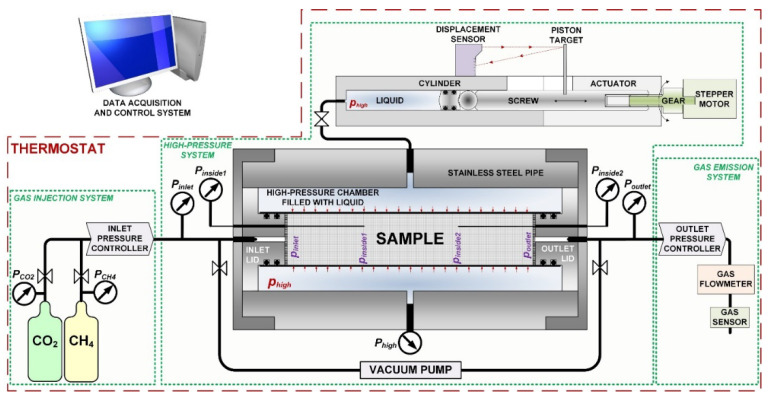
Schematic diagram of apparatus for testing filtration, sorption and CO_2_/CH_4_ exchange sorption processes under isobaric conditions on sorbent subjected to confining pressure.

The constructed apparatus for testing filtration, sorption and CO_2_/CH_4_ exchange sorption processes under isobaric conditions on sorbent subjected to confining pressure and for measuring the effect of these processes on changes in the sample volume consisted of three systems (Figure 1):

- A high-pressure system;
- A gas injection system;
- A gas emission system.

To ensure the isothermal measurement conditions, all of the systems were placed in a Q-Cell 1400 (Pol-Lab) thermostatic cabinet. The operation of the equipment was controlled by means of a data acquisition and control system; a USB-1616HS-2 (Measurement Computing) converter card connected to a computer with DasyLab software.

### 2.1. High-Pressure System

**Figure 2:**
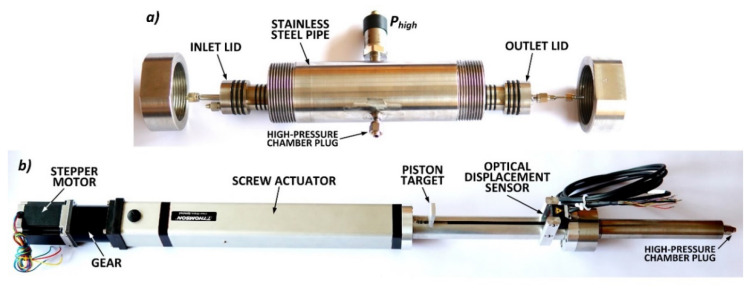
Photos of the high-pressure system elements: (**a**) high-pressure chamber; (**b**) mechanical actuator.

The high-pressure system was the main construction element and body of the device. In this system, filtration and gas sorption processes in a sample loaded with confining pressure were measured. This system consisted of (Figure 2):

1. A high-pressure chamber filled with liquid (distillated water) made of a stainless steel, thick-walled pipe with an internal diameter of 4.0 cm, an external diameter of 7.0 cm and a length of 24.0 cm;
2. A mechanical actuator consisting of a stepper motor with a built-in planetary gear 57HSG82-3004A14-B34 1:16 (discotech), which through a PC40LX266B05-0200XM1 (Thomson) precision linear actuator drove an M10 screw connected to the piston (with a diameter of 1 cm), thus regulating the liquid pressure in the high-pressure chamber. The liquid pressure was measured with a pressure transducer S-10 (WIKA) with a measuring range of 40 MPa and a measuring error of 0.25% of the full scale;
3. A sample volume measurement sub-system consisting of a laser displacement sensor optoNCDT ILD1320-100 (Micro-Epsilon) with a measuring range of 10.0 cm and an uncertainty of 0.12% of the full scale recording the screw movement;
4. A subsystem with a sorbent sample, sealed from the outside with a Teflon jacket and lids at both ends, which was placed in a high-pressure chamber. The outer diameter of the sample was approximately 3.0 cm and its length was about 15.0 cm. The pressure at the inlet, outlet and inside the sample was measured using the $P_{inlet}$, $P_{outlet}$, $P_{inside1}$ and $P_{inside2}$ pressure transducers S-10 (WIKA) with a measuring range of 1.6 MPa and a measuring error of 0.25% of the full scale.

The confining pressure on the sorbent sample was applied through the liquid that filled the high-pressure chamber. The sorbent volume changes resulting among others from the sorption-induced swelling were determined on the basis of the piston displacement measurement from the formula:

(1) $$\Delta V_{s}=\frac{S\cdot \Delta x}{V_{s}}\cdot 100\%$$

where:

$S\;\left[{\mathrm{cm}}^{2}\right]$ is the surface area of the screw cross section;

Δx [cm] is the screw displacement;

$V_{s}\;\left[{\mathrm{cm}}^{3}\right]$ is the original volume of the sample.

### 2.2. Gas Injection System

The inlet system served as a supply of CH_4_ and CO_2_ sorbates. The idea of its operation was based on the functioning of the manostat [58] and the manometric sorptomat [49] but the executive element was a precise gas pressure controller. This system ensured that the sorbate (CO_2_ or CH_4_) was injected under constant pressure into the sorbent sample. The gas injection system consisted of:

1. A CO_2_ cylinder with a volume of 500 cm^3^ and a pressure transducer S-10 (WIKA) with a measuring range of 2.5 MPa and a measuring error of 0.25% of the full scale;
2. A CH_4_ cylinder with a volume of 300 cm^3^ and a pressure transducer S-10 (WIKA) with a measuring range of 2.5 MPa and a measuring error of 0.25% of the full scale;
3. A gas inlet pressure controller SLA5810 (Brooks Instrument) with a regulation range of 0.1–1.6 MPa and a pressure stabilization precision of 0.12% of the full scale.

Based on the pressure drop in CO_2_ and CH_4_ cylinders, the amount of sorbed gas in the sorbent sample was determined using the ideal gas equation:

(2) $$n_{in}=\frac{\left(p_{0}-p_{1}\right)\cdot V_{c}}{m\cdot R\cdot T}\cdot V_{m}$$

where:

$n_{in}\left[\frac{{\mathrm{cm}}^{3}}{g}\right]$ is the amount of gas sorbed per gram of the sample;

$p_{0},\;p_{1}\;\left[\mathrm{Pa}\right]$ are the initial and final pressure in the sorbate cylinder;

$V_{c}\;\left[m^{3}\right]$ is the volume of the sorbate cylinder;

$V_{m}\left[\frac{{\mathrm{cm}}^{3}}{\mathrm{mol}}\right]$ is the molar volume of gas under normal conditions;

m[g] is the mass of the sample;

$R\left[\frac{J}{\mathrm{mol}\cdot K}\right]$ is the universal gas constant;

T[K] is the temperature.

### 2.3. Gas Emission System

The gas emission system ensured pressure regulation at the sample outlet as well as balancing and analyzing the gas that flowed out of the sorbent sample in filtration and CO_2_/CH_4_ exchange sorption measurements. This system consisted of the following elements connected in a series:

1. A gas outlet pressure controller SLA5820 (Brooks Instrument) with a regulation range of 0.1–1.0 MPa and a pressure stabilization precision of 0.12% of the full scale;
2. A gas flow meter SLA5860 (Brooks Instrument) with a measurement range of 0–5 cm^3^/min and a measurement precision of 1.0% of the full scale;
3. An infrared gas sensor Gascard NG (Edinburgh Sensors) for measuring the concentration of CH_4_ with a measuring range of 100% and measuring precision ± 2.0% of the full scale. Optionally, the same model of CO_2_ measurement card can be used or both cards at the same time if necessary.

The gas outlet pressure controller provided isobaric gas pressure conditions during the measurement. Based on the registration of the gas flow rate at the sample outlet measured with a flow meter, the amount of gas released from the sample can be determined using the equation:

(3) $$n_{out}=\overset{t_{1}}{\underset{t_{0}}{\int }}\frac{Q_{out}}{m}\cdot dt$$

where:

$t_{0},\;t_{1}\;\left[\min\right]$ are the initial and final time of the measurement;

$n_{out}\left[\frac{{\mathrm{cm}}^{3}}{g}\right]$ is the amount of gas desorbed per gram of sample;

$Q_{out}\;\left[\frac{{\mathrm{cm}}^{3}}{\min}\right]$ is the gas flow rate at the sample outlet.

## 3. Apparatus Tests

As part of the work, basic apparatus tests were carried out to check its correct operation and determine its specifications. These tests consisted of carrying out trial measurements of CH_4_ filtration processes, CH_4_ sorption and CO_2_/CH_4_ exchange sorption on a coal sample. All experiments were preceded by leakage tests of the measuring apparatus after the sample was installed in it. All tests were carried out under isothermal conditions at 303.15 K.

### 3.1. Research Material

**Figure 3:**
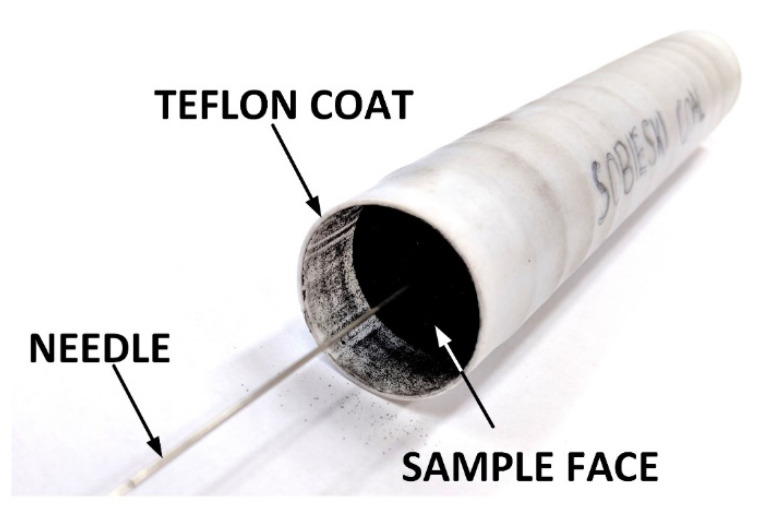
Sample of coal briquette prepared for tests.

The research material used for the tests was obtained from the Budryk mine from the Upper Silesian Coal Basin in Poland. The sample was a properly prepared coal briquette with a diameter of 3.0 cm, a length of 14.4 cm and a weight of 136.2 g (Figure 3). The briquette was created by pressing a granular dry sample of grain size below 0.05 cm without any binder on a hydraulic press. The pressing pressure during the sample preparation was 40 MPa.

In order to protect the sample from liquid that transferred to confining pressure on it during measurement, it was sealed from the outside with a thin (less than 0.1 cm thick) Teflon coat and lids at both ends. In order to register changes in the gas pressure inside the sample, needles reaching a depth of about 5 cm were placed at the inlet and outlet of the sample (Figure 3).

The sample prepared in such a way for tests was placed in the high-pressure chamber of the measuring apparatus. Distilled water was then injected into the chamber as a confining pressure medium. The tests of the device were carried out with different values of confining pressure.

### 3.2. Methane Filtration Tests

**Figure 4:**
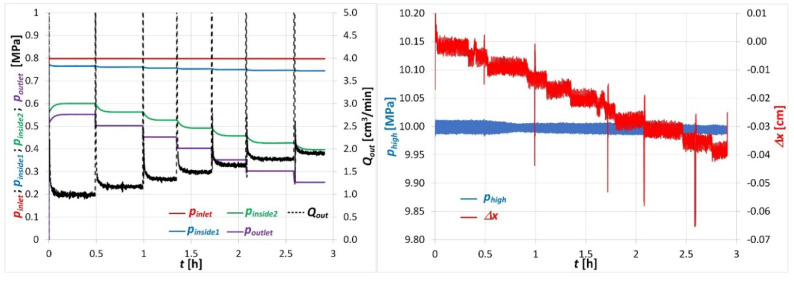
Courses of changes of some parameters registered during the filtration of CH_4_ through a coal briquette loaded with a confining pressure of 10 MPa.

The tests of gas filtration through the coal briquette were carried out with the use of CH_4_ and at a confining load exerted on the sample equal to 10 MPa. The CH_4_ pressure at the sample inlet was stabilized at 0.8 MPa. The pressure at the sample outlet was stabilized at 0.55 MPa, 0.50 MPa, 0.45 MPa, 0.40 MPa, 0.35 MPa, 0.30 MPa and 0.25 MPa and each pressure was maintained until the *Q_out_* flow rate stabilized. The registered changes of the pressure values of *p_inlet_*, *p_inside1_*, *p_inside2_*, *p_outlet_* and the corresponding changes of flow rate *Q_out_* at the sample outlet are shown in Figure 4.

As it can be seen in Figure 4, decreasing the outlet pressure *p_outlet_*, with a constant pressure *p_inlet_* at the sample inlet, resulted in an increase in the CH_4_ flow rate *Q_out_* at the sample outlet. Simultaneously, pressures *p_inside1_*, *p_inside2_* inside the coal briquette also changed proportionally to the *p_outlet_* pressure. The *p_inside1_* dropped slightly, while *p_inside2_* dropped much more dynamically about 0.05 MPa with each step of the *p_outlet_* pressure change. The registered changes of CH_4_ pressures occurring in the sample and the gas flow rate at the sample outlet were typical changes accompanying the process of gas filtration through a porous medium [23]. Thus, it can be concluded that the functioning of the gas pressure sensors and flow meter in the apparatus were correct and they ensured a temporal and spatial observation of the distribution of the pressures and gas flow rate in the tested sample.

When analyzing the course of changes in the confining pressure *p_high_* around the sample, it could be seen that this regulation was performed with a high precision of about ±0.02 MPa (which was ±0.2% of the regulated value). This stabilization was maintained throughout the test period (3 h), which could be considered as the correct operation of the regulator.

Based on the registration of the piston movement *Δx*, which exerts pressure on the liquid filling the high-pressure chamber, changes in the sample volume in the filtration measurement cycle could be determined. During the test it was observed that a change in pressure *p_outlet_* at the sample outlet was followed by the movement of the piston. The registered displacement of the piston in the negative direction indicated that the sample decreased in volume and thus shrank as the mean value of the CH_4_ pressure in the sample decreased. It was also observed that the fluctuation of the piston displacement registration was due among other things to the operation of the high-pressure regulator of the liquid, which exerted a confining pressure on the sample. The accuracy of the piston displacement measurement was about ±0.002 cm, which, with the piston surface equal to 0.785 cm^2^, corresponded to the precision of volume changes registration equal to ±0.001 cm^3^. With a sample volume of about 100 cm^3^, the precision of measuring changes in the sample volume was 0.1%.

### 3.3. Sorption Tests

**Figure 5:**
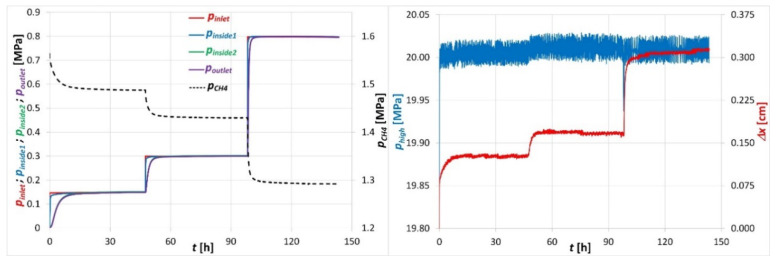
Courses of changes of some parameters registered during the sample saturation with CH_4_.

Laboratory tests of CO_2_/CH_4_ exchange sorption are usually preceded by saturating the sample with CH_4_ to the sorption equilibrium state at specified pressure and temperature conditions. On the constructed apparatus, the CH_4_ sample saturation tests were performed with the gas emission system cut off, which was ensured by closing the outlet pressure controller (Figure 1) during the CH_4_ saturation step. Sorption tests were carried out under isobaric conditions and consisted of an injection of CH_4_ at 0.15, 0.30 and 0.80 MPa into a sample loaded with a confining pressure of 20 MPa. The confining pressure of 20 MPa corresponds to the overburden pressure at a depth of about 800 m, from which the Budryk coal sample was collected. Figure 5 presents the pressure changes of *p_inlet_*, *p_insde1_*, *p_inside2_*, *p_outlet_* and *p_CH4_*, as well as the corresponding piston displacement *Δx*, during the sample saturation with CH_4_.

CH_4_ sorption tests were performed at three different sorption equilibrium pressures and the saturation time at each pressure was about 50 h until the sorption equilibrium was reached. CH_4_ was injected under constant pressure into the sample inlet and then it flowed along the sample, hence the values of subsequent *p_inlet_*, *p_insde1_*, *p_inside2_* and *p_outlet_* pressures increased to a constant value with an increasing delay.

The registered drop in the *p_CH4_* pressure in the supply cylinder during the sample saturation with CH_4_ was exponential at each saturation pressure. This drop was proportional to the CH_4_ sorption process taking place in the coal sample.

Based on the analysis of the *p_high_* confining pressure changes registration, it could be seen that, as in the filtration test, the stabilization of this pressure was carried out with a high precision of ±0.02 MPa during the saturation test lasting 150 h. The displacement sensor registered the displacement *Δx* of the piston, which was directly proportional to the changes in the volume of the sample and proportional to the CH_4_ sorption in the sample. This was the result of sample swelling due to CH_4_ sorption.

**Figure 6:**
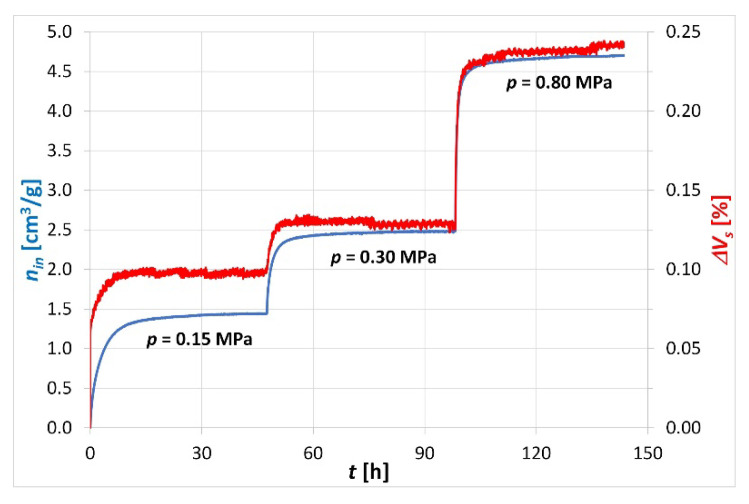
Amount of sorbed CH_4_ and corresponding changes in sample volume during CH_4_ saturation of the sample at a confining pressure of 20 MPa.

Based on the registered courses of piston displacement *Δx* and pressure *p_CH4_* changes in the CH_4_ cylinder during the sample saturation, the changes in sample volume *ΔV_S_* and the amount of CH_4_ sorbed per gram of sample *n_in_* could be determined from Formulas (1) and (2) (Figure 6).

In the CH_4_ sorption test carried out at three different sorption equilibrium pressures, the sample sorption capacities of 1.44, 2.48 and 4.70 cm^3^/g were obtained, respectively. A relative increase in sample volume of 0.10%, 0.13% and 0.24% corresponded to sorption measurements. Based on the obtained results of a CH_4_ sorption measurement on a sample subjected to confining pressure, it was found that the constructed apparatus worked correctly.

### 3.4. Exchange Sorption Tests

**Figure 7:**
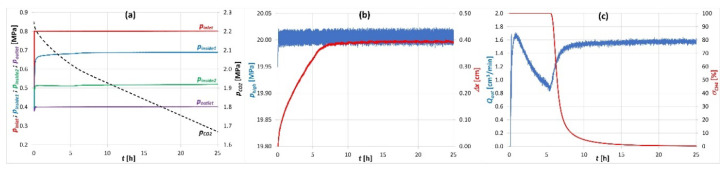
Courses of changes of some parameters registered during the CO_2_/CH_4_ exchange sorption processes under isobaric conditions on sorbent subjected to confining pressure; (**a**) pressure distribution of *p_inlet_*, *p_insde1_*, *p_inside2_* and *p_outlet_* along the briquette, and *p_CO_2__* pressure changes in the supply cylinder; (**b**) the confining pressure *p_high_* and piston displacement *Δx*; (**c**) the gas flow rate *Q_out_* and CH_4_ concentration *σ_CH4_* at the sample outlet.

The CO_2_/CH_4_ exchange sorption tests were performed under isobaric conditions and consisted -in the injection of CO_2_ into the sample saturated with CH_4_. Prior to the CO_2_/CH_4_ exchange sorption test, the coal sample was saturated with CH_4_ at 0.40 MPa until sorption equilibrium was reached. CO_2_ was then injected into the sample inlet at a pressure of 0.80 MPa. The saturation pressure of CH_4_ equal to 0.4 MPa corresponded to the methane pore pressure in the coal seam from which the coal sample was taken. The confining pressure exerted on the sample during the test was stabilized at a constant level of 20 MPa. Courses of registered parameters’ changes during the exchange sorption test are presented in Figure 7.

The measurement of CO_2_/CH_4_ exchange sorption is an extremely complicated study due to the large number of processes taking place in the coal sample. Hence, the number of parameters necessary to be registered during the measurement is large. Changes in pressure distribution of *p_inlet_*, *p_insde1_*, *p_inside2_* and *p_outlet_* along the briquette accompanying exchange sorption are shown in Figure 7a. With constant pressures at the inlet (*p_inlet_* = 0.80 MPa) and outlet (*p_outlet_* = 0.40 MPa) of the coal briquette, the pressures inside the sample reached *p_insde1_* = 0.69 MPa and *p_inside2_* = 0.52 MPa. During the exchange sorption, the *p_CO2_* pressure in the supply cylinder decreased. The confining pressure during the exchange sorption measurement was stabilized at 20 MPa with a precision of ± 0.02 MPa (Figure 7b).

Due to the multiplicity of processes occurring during the exchange sorption, it was necessary to start analyzing the results on the basis of changes in the concentration of the CO_2_-CH_4_ mixture (*σ_CO2_*, *σ_CH4_*) at the sample outlet (Figure 7c). As it can be seen in the graph, pure CH_4_ was registered at the coal outlet for the time of 5.6 h from the start of the measurement (1st phase: *σ_CH4_* = 100%). The concentration of CH_4_ then started to decrease intensively to reach 10% after 8.3 h from the start of the measurement (2nd phase: 100% < *σ_CH4_* < 10%). From then on, the intensity of the CH_4_ concentration decrease slowed down (3rd phase: *σ_CH4_* < 10%). When analyzing the gas flow rate *Q_out_* at the sample outlet it could be seen that in the 1st phase, the CH_4_ output decreased from 1.7 cm^3^/min to 0.8 cm^3^/min. In the 2nd phase, the flow rate of the CO_2_-CH_4_ mixture increased from 0.8 cm^3^/min to 1.6 cm^3^/min and in the 3rd phase, the flow rate of CO_2_ at the sample outlet was constant and amounted to 1.6 cm^3^/min. The displacement sensor registered swelling of the sample in the 1st phase. In the subsequent phases, the sample did not change its volume.

All of the changes in parameters during the CO_2_/CH_4_ exchange sorption process were related to the sorption exchange zone moving along the briquette [43,55]. In the first phase, CO_2_ was sorbed in the coal, thus displacing CH_4_. The exchange process zone moved from the sample inlet to the sample outlet. In the 2nd phase, the exchange zone reached the outlet of the briquette so that the CH_4_ concentration decreased and the CO_2_ concentration increased. In the 3rd phase, CH_4_ was almost entirely displaced by CO_2_ and there was mainly a process of CO_2_ filtration in the meso- and macropore space of the coal briquette. All observed phenomena were confirmation of the correct operation of the apparatus during the measurement of CO_2_/CH_4_ exchange sorption on a coal sample under confining pressure conditions [43,44].

**Figure 8:**
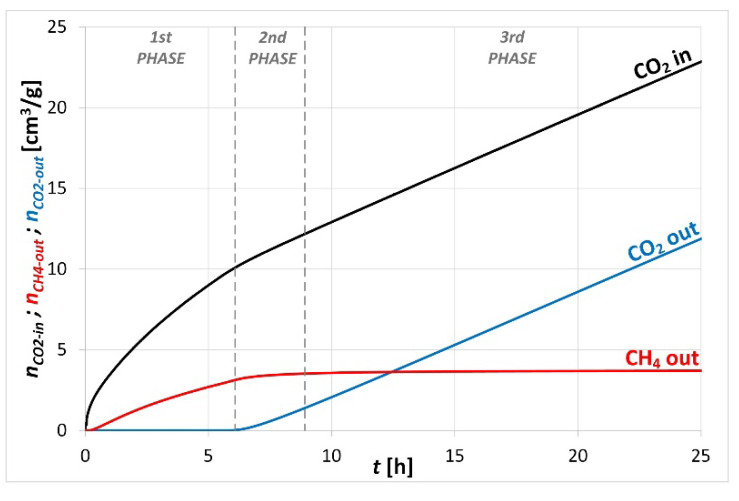
Balance of CO_2_/CH_4_ exchange sorption processes under isobaric conditions on a sorbent subjected to confining pressure.

Based on the results recorded during the CO_2_/CH_4_ exchange sorption test it was possible to determine the balance of the exchange sorption process. An example of the CO_2_/CH_4_ exchange sorption balance is shown in Figure 8.

As it can be seen from the example of the conducted balance of CO_2_/CH_4_ exchange sorption (Figure 8), in the 1st phase, when a competitive sorption process took place in the sample consisting in the displacement of CH_4_ particles by preferentially sorbed CO_2,_ the intensity of the CO_2_ injection was the highest. In this phase, pure CH_4_ flowed out at the outlet of the sample. In the 2nd phase, when the CO_2_/CH_4_ exchange zone reached the sample outlet, the intensity of CO_2_ injection into the sample inlet decreased. In the 3rd phase, the changes in the amount of injected CO_2_ and the amount of CO_2_ flowing out of the sample were the same and the amount of CH_4_ at the outlet did not increase, which suggested that it was displaced during the exchange sorption process.

The presented data showing the course of CO_2_/CH_4_ exchange sorption are necessary to assess the effectiveness and applicability of the CO_2_-ECBM technology for this particular coal [44]. A great advantage of the constructed apparatus is the possibility to perform tests under confining pressure conditions corresponding to the in situ conditions.

## 4. Conclusions

Issues related to the design and development of new solutions for testing CO_2_-ECBM technologies are very important in the context of laboratory analysis of the potential of the method, its efficiency and applicability. An innovative apparatus for testing filtration, sorption and CO_2_/CH_4_ exchange sorption processes under isobaric conditions on sorbent subjected to confining pressure was designed and constructed at the Strata Mechanics Research Institute of the Polish Academy of Sciences. Tests of filtration, sorption and CO_2_/CH_4_ exchange sorption carried out on the constructed apparatus and performed on a sample of coal briquette subjected to a confining pressure of 10 MPa and 20 MPa showed the following:

- The regulation of the confining pressure is possible in the range of 0.1–40 MPa, with the stabilization precision equal to ± 0.02 MPa, although in the tests the maximum confining pressure was 20 MPa;
- The pressure at the inlet and outlet of the sample can be regulated in the range of 0.1–1.6 MPa and 0.1–1.0 MPa, respectively, with pressure stabilization precision of 0.12% of the full scale and in the conducted tests the inlet and outlet pressures were regulated in the range of 0.4–0.8 MPa;
- The measurement range of the sample volume changes is 0–7.85 cm^3^ and the measurement precision is ± 0.001 cm^3^, which corresponds to 7.4% for the sample size used in the tests performed;
- The measurement range of filtration is limited by the measuring range of the gas flow meter and is 0–5 cm^3^/min and a measurement precision of 1.0% of the full scale;
- The CH_4_ and CO_2_ sorption measurement range depends on the sorption measurement pressure, limited by the CH_4_ and CO_2_ supply cylinders volume, the pressure sensor measurement range and the mass of the sorbent sample. With a sample mass of about 100 g and sorption pressure of 0.1 MPa, the CH_4_ and CO_2_ sorption measurement ranges are about 0–72 cm^3^/g and 0–120 cm^3^/g respectively.

The results of the tests presented in the article confirmed the correct functioning of the constructed apparatus. Currently, the apparatus is used to carry out detailed studies of CO_2_/CH_4_ exchange sorption on coal samples subjected to confining pressure. The aim of this study is to assess coal as a reservoir for CO_2_ storage and its applicability to CO_2_-ECBM technology. The results of these detailed studies performed on the constructed apparatus will be published soon.

The possibilities of applying this apparatus are very wide. The apparatus can also work in case of other solid materials/sorbents/rocks and other gases and their mixtures.

## 5. Patents

The unique characteristics of the apparatus were specified in a patent application filed with the Polish Patent Office (Patent application number: 433748; Authors: Kudasik M., Skoczylas N., Pajdak A.: Device for testing sorption processes under isobaric conditions on sorbent subjected to quasi-hydrostatic loading and for measuring the effect of these processes on changes in the volume of sorbent. Submitted: 27 April 2020).

## References

[1] NASA: Climate Change and Global Warming. https://climate.nasa.gov/evidence/.

[2] Gray I. (1987). Reservoir Engineering in Coal Seams: Part 1-The Physical Process of Gas Storage and Movement in Coal Seams. SPE Reserv. Eng..

[3] Liu J., Spiers C., Peach C.J., Vidal-Gilbert S. (2016). Effect of lithostatic stress on methane sorption by coal: Theory vs. experiment and implications for predicting in-situ coalbed methane content. Int. J. Coal Geol..

[4] Czerw K., Baran P., Zarębska K. (2017). Application of the stretched exponential equation to sorption of mine gases and sorption induced swelling of bituminous coal. Int. J. Coal Geol..

[5] Skiba M., Młynarczuk M. (2018). Identification of macerals of the inertinite group using neural classifiers, based on selected textural features. Arch. Min. Sci..

[6] Skoczylas N., Pajdak A., Kudasik M., Braga L.T.P. (2020). CH_4_ and CO_2_ sorption and diffusion carried out in various temperatures on hard coal samples of various degrees of coalification. J. Nat. Gas Sci. Eng..

[7] Larsen J.W. (2004). The effects of dissolved CO_2_ on coal structure and properties. Int. J. Coal Geol..

[8] Karacan C.Ö. (2007). Swelling-induced volumetric strains internal to a stressed coal associated with CO_2_ sorption. Int. J. Coal Geol..

[9] Zarębska K., Ceglarska-Stefańska G. (2008). The change in effective stress associated with swelling during carbon dioxide sequestration on natural gas recovery. Int. J. Coal Geol..

[10] Day S., Fry R., Sakurovs R. (2012). Swelling of coal in carbon dioxide, methane and their mixtures. Int. J. Coal Geol..

[11] Baran P., Zarębska K., Bukowska M. (2015). Expansion of Hard Coal Accompanying the Sorption of Methane and Carbon Dioxide in Isothermal and Non-isothermal Processes. Energy Fuels.

[12] Zhang Y., Lebedev M., Sarmadivaleh M., Barifcani A., Rahman T., Iglauer S. (2016). Swelling effect on coal micro structure and associated permeability reduction. Fuel.

[13] Han F., Chen G., Liu Z., Yang J. (2017). Correlation of swelling and sorption properties of block coal sample. Fuel.

[14] Zang J., Wang K. (2017). Gas sorption-induced coal swelling kinetics and its effects on coal permeability evolution: Model development and analysis. Fuel.

[15] Pone J.D.N., Halleck P.M., Mathews J.P. (2010). 3D characterization of coal strains induced by compression, carbon dioxide sorption, and desorption at in-situ stress conditions. Int. J. Coal Geol..

[16] Majewska Z., Majewski Z., Ziętek J. (2013). Swelling and acoustic emission behaviour of unconfined and confined coal during sorption of CO. Int. J. Coal Geol..

[17] Pone J.D.N., Halleck P.M., Mathews J.P. (2009). Sorption Capacity and Sorption Kinetic Measurements of CO_2_ and CH_4_ in Confined and Unconfined Bituminous Coal. Energy Fuels.

[18] Pone J.D.N., Hile M., Halleck P.M., Mathews J.P. (2009). Three-dimensional carbon dioxide-induced strain distribution within a confined bituminous coal. Int. J. Coal Geol..

[19] Jikich S.A., McLendon R., Seshadri K., Irdi G., Smith D.H. (2009). Carbon dioxide transport and sorption behaviour in confined coal for carbon sequestration. SPE Reserv. Eval. Eng..

[20] Wang G., Wei X., Wang K., Massarotto P., Rudolph V. (2010). Sorption-induced swelling/shrinkage and permeability of coal under stressed adsorption/desorption conditions. Int. J. Coal Geol..

[21] Hol S., Peach C.J., Spiers C. (2011). Applied stress reduces the CO_2_ sorption capacity of coal. Int. J. Coal Geol..

[22] Hol S., Spiers C. (2012). Competition between adsorption-induced swelling and elastic compression of coal at CO_2_ pressures up to 100MPa. J. Mech. Phys. Solids.

[23] Kudasik M. (2019). Investigating Permeability of Coal Samples of Various Porosities under Stress Conditions. Energies.

[24] Ceglarska-Stefańska G., Zarębska K. (2002). The competitive adsorption of CO_2_ and CH_4_ with regard to the release of methane from coal. Fuel Process. Technol..

[25] Mazumder S., Wolf K.H. (2008). Differential swelling and permeability change of coal in response to CO_2_ injection for ECBM. Int. J. Coal Geol..

[26] Czerw K., Zarębska K., Buczek B., Baran P. (2016). Kinetic models assessment for swelling of coal induced by methane and carbon dioxide sorption. Adsorption.

[27] Fulton P.F., Parente C.A., Rogers B.A., Shah N., Reznik A.A. (1980). A laboratory Investigation of Enhanced Recovery of Methane from Coal by Carbon Dioxide Injection.

[28] Reznik A., Singh P., Foley W. (1982). Enhanced Recovery of In Situ Methane by Carbon Dioxide Injection: An Experimental Feasibility Study.

[29] Clarkson C., Bustin R. (2000). Binary gas adsorption/desorption isotherms: Effect of moisture and coal composition upon carbon dioxide selectivity over methane. Int. J. Coal Geol..

[30] Krooss B.M., Van Bergen F., Gensterblum Y., Siemons N., Pagnier H.J.M., David P. (2002). High-pressure methane and carbon dioxide adsorption on dry and moisture-equilibrated Pennsylvanian coals. Int. J. Coal Geol..

[31] Busch A., Krooss B.M., Gensterblum Y., van Bergen F., Pagnier H.J.M. (2003). High-pressure adsorption of methane, carbon dioxide and their mixtures on coals with a special focus on the preferential sorption behaviour. J. Geochem. Explor..

[32] Busch A., Gensterblum Y., Krooss B.M., Siemons N. (2006). Investigation of high-pressure selective adsorption/desorption behaviour of CO_2_ and CH_4_ on coals: An experimental study. Int. J. Coal Geol..

[33] Majewska Z., Ceglarska-Stefańska G., Majewski S., Ziętek J. (2009). Binary gas sorption/desorption experiments on a bituminous coal: Simultaneous measurements on sorption kinetics, volumetric strain and acoustic emission. Int. J. Coal Geol..

[34] Baran P., Broś M., Nodzeński A. (2010). Studies on CO_2_ sorption on hard coal in the near-critical area with regard to the aspect of sequestration. Arch. Min. Sci..

[35] Yu H., Jing R., Wang P., Chen L., Yang Y. (2014). Preferential adsorption behaviour of CH 4 and CO 2 on high-rank coal from Qinshui Basin, China. Int. J. Min. Sci. Technol..

[36] Mazumder S., Wolf K.H.A.A., Van Hemert P., Busch A. (2008). Laboratory Experiments on Environmental Friendly Means to Improve Coalbed Methane Production by Carbon Dioxide/Flue Gas Injection. Transp. Porous Media.

[37] Yu H., Yuan J., Guo W., Cheng J., Hu Q. (2008). A preliminary laboratory experiment on coalbed methane displacement with carbon dioxide injection. Int. J. Coal Geol..

[38] Wolf K.H.A.A., Siemons N., Bruining J. (2004). Multiphase flow experiments in order to understand the behavior of (partly) saturated coals as a gas reservoir: Examples. Geol. Belg..

[39] Jessen K., Tang G.-Q., Kovscek A.R. (2007). Laboratory and Simulation Investigation of Enhanced Coalbed Methane Recovery by Gas Injection. Transp. Porous Media.

[40] Shi J.-Q., Mazumder S., Wolf K.-H., Durucan S. (2008). Competitive Methane Desorption by Supercritical CO_2_ Injection in Coal. Transp. Porous Media.

[41] Liang W., Zhao Y., Wu D., Dusseault M.B. (2011). Experiments on Methane Displacement by Carbon Dioxide in Large Coal Specimens. Rock Mech. Rock Eng..

[42] Bhowmik S., Dutta P. (2011). Investigation into the Methane Displacement Behavior by Cyclic, Pure Carbon Dioxide Injection in Dry, Powdered, Bituminous Indian Coals. Energy Fuels.

[43] Dutka B., Kudasik M., Topolnicki J. (2012). Pore pressure changes accompanying exchange sorption of CO_2_/CH_4_ in a coal briquette. Fuel Process. Technol..

[44] Dutka B., Kudasik M., Pokryszka Z., Skoczylas N., Topolnicki J., Wierzbicki M. (2013). Balance of CO_2_/CH_4_ exchange sorption in a coal briquette. Fuel Process. Technol..

[45] Davis D., Oudinot A., Sultana A., Reeves S. (2004). CoalSeq 2.2: A Screening Model for ECBM Recovery and CO_2_ Sequestration in Coal. Topical Report and Users Manual—ARI and US Department of Energy. www.coal-seq.com.

[46] Gawor M., Skoczylas N. (2013). Sorption Rate of Carbon Dioxide on Coal. Transp. Porous Media.

[47] Skoczylas N. (2015). Determining the gas permeability coefficient of a porous medium by means of the bubble-counting flow meter. Meas. Sci. Technol..

[48] Skoczylas N., Kudasik M., Wierzbicki M., Murzyn T. (2015). New Instruments and Methods for Analysing the Coal-Methane System. Stud. Geotech. Mech..

[49] Kudasik M. (2016). The manometric sorptomat–an innovative volumetric instrument for sorption measurements performed under isobaric conditions. Meas. Sci. Technol..

[50] Kudasik M. (2017). Results of comparative sorption studies of the coal-methane system carried out by means of an original volumetric device and a reference gravimetric instrument. Adsorption.

[51] Kudasik M., Skoczylas N. (2017). Analyzer for measuring gas contained in the pore space of rocks. Meas. Sci. Technol..

[52] Pajdak A., Godyń K., Kudasik M., Murzyn T. (2017). The use of selected research methods to describe the pore space of dolomite from copper ore mine, Poland. Environ. Earth Sci..

[53] Kudasik M., Pajdak A., Skoczylas N. (2018). The Validation Process of the Method of Balancing Gas Contained in the Pore Space of Rocks via Rock Comminution. Arch. Min. Sci..

[54] Skoczylas N., Wierzbicki M., Kudasik M. (2018). A simple method for measuring basic parameters of the coal—methane system under mining conditions. J. Min. Sci..

[55] Topolnicki J., Kudasik M., Dutka B. (2013). Simplified model of the CO_2_/CH_4_ exchange sorption process. Fuel Process. Technol..

[56] Pajdak A., Kudasik M., Skoczylas N., Wierzbicki M., Braga L.T.P. (2019). Studies on the competitive sorption of CO_2_ and CH_4_ on hard coal. Int. J. Greenh. Gas Control..

[57] Pajdak A., Skoczylas N., Dębski A., Grzegorek J., Maziarz W., Kudasik M. (2019). CO_2_ and CH_4_ sorption on carbon nanomaterials and coals—Comparative characteristics. J. Nat. Gas Sci. Eng..

[58] Kudasik M., Skoczylas N., Sobczyk J., Topolnicki J. (2010). Manostat—an accurate gas pressure regulator. Meas. Sci. Technol..

